# Different Brain Network Activations Induced by Modulation and Nonmodulation Laser Acupuncture

**DOI:** 10.1155/2011/951258

**Published:** 2010-09-01

**Authors:** Chang-Wei Hsieh, Jih-Huah Wu, Chao-Hsien Hsieh, Qwa-Fun Wang, Jyh-Horng Chen

**Affiliations:** ^1^Department of Photonic and Communication Engineering, Asia University, Taichung 41354, Taiwan; ^2^Department of Biomedical Engineering, Ming Chuan University, Taoyuan 33347, Taiwan; ^3^Interdisciplinary MRI/MRS Laboratory, Department of Electrical Engineering, National Taiwan University, Taipei 10617, Taiwan; ^4^Neurobiology and Cognitive Science Center, National Taiwan University, Taipei 10617, Taiwan; ^5^School of Post-Baccalaureate Chinese Medicine, China Medical University, Taichung 40402, Taiwan

## Abstract

The aim of this study is to compare the distinct cerebral activation with continued wave (CW) and 10 Hz-modulated wave (MW) stimulation during low-level laser acupuncture. Functional magnetic resonance imaging (fMRI) studies were performed to investigate the possible mechanism during laser acupuncture stimulation at the left foot's yongquan (K1) acupoint. There are 12 healthy right-handed volunteers for each type of laser stimulation (10-Hz-Modulated wave: 8 males and 4 females; continued wave: 9 males and 3 females). The analysis of multisubjects in this experiment was applied by random-effect (RFX) analysis. In CW groups, significant activations were found within the inferior parietal lobule, the primary somatosensory cortex, and the precuneus of left parietal lobe. Medial and superior frontal gyrus of left frontal lobe were also aroused. In MW groups, significant activations were found within the primary motor cortex and middle temporal gyrus of left hemisphere and bilateral cuneus. Placebo stimulation did not show any activation. Most activation areas were involved in the functions of memory, attention, and self-consciousness. The results showed the cerebral hemodynamic responses of two laser acupuncture stimulation modes and implied that its mechanism was not only based upon afferent sensory information processing, but that it also had the hemodynamic property altered during external stimulation.

## 1. Introduction

Acupuncture, one of the oldest medical treatments, was considered an ancient Chinese method to cure disease and reduce pain. However, an archaeological report suggested another origin. The tattoo locations on the Stone Age body found in the Alps correspond closely with Chinese acupuncture points and furthermore correspond to points used to treat lumbar and leg-joint arthritis and abdominal disorders [1]. Even so, after the passing of thousands of years, acupuncture-related treatments are not well developed in Europe. In ancient China, acupuncture is not only a folk therapy, but also incorporates the metaphysical theory of “Qi,” a supposed vital energy that runs through hypothesized channels, called “meridians.” In recent decades, the international community's related programs and researches have been increasing, resulting in the traditional techniques gradually gaining acceptance worldwide. Current researches concerning acupuncture can be classified into three kinds: the first is to revalidate the effect of specific acupuncture points; the second is to understand pain processing; the third is to modulate the pain with acupuncture [2].

Functional magnetic resonance imaging (fMRI), which can be used to observe the response of the human brain with the advantages of noninvasiveness and nonradiation, has become an important scientific tool, in particular for those senior human cognitive neuropsychological researches that cannot be done on animals. Functional MRI could show the signal change while the hemoglobin status is changing. Hemoglobin is diamagnetic when oxygenated but paramagnetic when deoxygenated. The magnetic resonance (MR) signal of blood is slightly different depending on the level of oxygenation. Therefore, fMRI research uses the property of blood-oxygen-level dependent (BOLD) as the method for determining where activities occurred in the brain as the result of various experiences. Functional MRI is an effective tool to observe the human brain's response of acupuncture stimulation. The application of the fMRI technique for acupuncture study has been used since the mid-1990s [3], and two important types of acupuncture experiments—needle and electrical stimulations—are performed [4–11].

Studies using fMRI have also investigated how acupuncture modulates well-characterized pain stimuli, nonpain somatosensation, and even resting brain function [2]. As time has passed, the technology used to stimulate acupuncture points has progressed from stone to metal and more recently to electroacupuncture; the most recent technologic development has been the introduction of laser acupuncture, defined as the stimulation of traditional acupuncture points with low-intensity, nonthermal laser irradiation. Inspecting its therapeutic efficacy, Whittaker [12] considers the depth of laser energy transmission the most important determinant. Among multiplicity stimulation methods, using low-level laser therapy (LLLT) is the easiest way to design a pure placebo acupuncture experiment in which subjects were not to distinguish placebo acupuncture from verum acupuncture [13–16].

Many papers investigate the biological models of acupuncture. As Shang [17] proposed, the mechanism of acupuncture may concern the nervous system, the circulatory system and other physiological systems. Dhond et al. [18] recently found that following verum, but not sham, acupuncture, there was increased resting functional connectivity between specific brain areas and the default mode network (DMN), a network of brain regions more active during a nontask processing state. Somehow, those studies pertaining to resting functional connectivity have a key idea similar to the meridian system of acupuncture in traditional Chinese medicine. The so-called functional connectivity is to observe that these brain regions are thought to possess dynamic, synchronized oscillations. Meridian systems comprise collected acupoints having meridians with like properties. A recent study from Wu et al. [19] indicates that functional connectivity has distinct frequency-specific features in the resting-state fMRI signal within these functional networks. When Ulett and Han et al. explain the mechanisms of acupuncture, they also focus on the different frequencies that stimulation produces with the release of different neuropetides [20, 21]. Furthermore, both Zhang et al. [22] and Napadow et al. [11] using fMRI to observe the brain network activation were mediated by electroacupuncture at different frequencies. The resting state of brain functional connectivity has the distinct frequency-specific features, with the similarity that the different frequencies of EA stimulation create different brain-area activation.

Siedentopf et al. [13, 15], using fMRI, observe the brain activations ipsilateral to the body side to which laser acupuncture is applied. They infer that the acupuncture is not solely based upon the processing of afferent neural somatosensory information. The other evidence shows that the low-level laser irradiation can promote local blood flow, and is the most possible path of the therapeutic mechanism [12, 16, 23]. Thus, using low-level laser as the stimulation source of acupuncture has the benefit of focusing the physiological systems involved in this study.

Despite many published studies concerning LLLT, no work has been done comparing different modulated stimulation on the same acupoint in one experiment. This work has been investigated with EA [11, 20–22]. This paper aims to inspect whether laser with a mechanism distinct from electrical stimulation would produce a similar phenomenon. According to the ancient TCM book, Nan Jing (Classic of Difficulties), the acupoint, K1 (Yongquan), is one of the Well (Jing) points. In clinical practice, stimulation K1 has the effects of curing insomnia, poor memory and mania, and so forth. The acupoint K1 is an important acupoint for humans, but less attention has been given to it in recent researches. From Whittaker's review of 30 laser acupuncture articles [12], modulated and nonmodulated treatments are often used alternately in these related studies. According to Han's report [21], opioid peptides and opioid receptors involved in analgesia are elicited by electroacupuncture of different frequencies. In low frequency (less than decade Hz), enkephalins release more efficiency; on the contrary in high frequency (up above decade Hz), dynorphins release more efficiency. At 10 Hz, the intermediate frequency, there is a mediate secretion at both of those neuropeptides. Further, Lee's study of 10 Hz external stimulation by sound or flash light can easily induce the somatic sensory so-called deqi in human** beings [24]. **Deqi is a big issue for acupuncture treatment of TCM. That is why we compare the 10-Hz-modulated Laser therapy with continued one. Therefore, we use LLLT to stimulate K1 (Yongquan) acupoint and observe the related human activations of the human brain.

## 2. Method and Materials

There are 12 healthy right-handed volunteers for each type of laser stimulation (10-Hz-modulated wave, MW: 8 males and 4 females; continued wave, CW: 9 males and 3 females), aged 27.9 ± 5.8 (mean ± S.D.) years for the MW pattern, and aged 28.6 ± 6.5 (mean ± S.D.) years for the CW pattern. No subject had a history of psychiatric or neurological disorders. Subjects were told they would experience laser acupuncture, but they were not told whether it was at verum or placebo laser stimulation for these two laser stimulation types. The acupuncture point, Yongquan (Kidney 1, K1), in this study was on the left foot. To avoid conditioning and long-lasting effects of laser acupuncture, placebo laser acupuncture was always executed before the verum experiment. Placebo acupuncture was used to control artificially induced brain activity caused by the setup of the experiment and to exclude any effects of anticipation. The subjects were told that the placebo and laser stimulation would be presented in a random order. Subjects were not able to recognize placebo acupuncture or verum acupuncture because the verum stimulation and the dummy stimulation differed only by switching the laser light on or off. And they were taught to lie easily with their hands on the abdomen, to close their eyes, and not to engage in any specific mental activity. The light in the scan room was turned off during the fMRI measurement, and there was no acoustic stimulation besides the scanner noise. Each one was asked to provide written consent, and the study protocol was approved by the Research Ethics Committee of National Taiwan University Hospital.

Experiments were performed on a Bruker MEDSPEC 3T system (Bruker, Ettlingen, Germany) with a quadrature head coil. Foam padding and a flexible belt fastened on the subject's head were used to restrain head motions during all image acquisitions. Images were acquired using gradient-echo echo planar imaging (EPI) with field of view 240 mm × 240 mm, matrix size of 64 × 64, 5 mm slice thickness with no gap, TE of 30 ms, and TR of 4 seconds. We used low-level laser diode that operates with continued wave laser beam (30 mW output, 808 nm wavelength, and 1 cm radius profile). In the configuration of this experiment setup, we used the GRASS S44 Stimulator (GRASS TECHNOLOGIES, USA) at settings of 5 V and 10 Hz 50% duty cycle. The stimulator was outside the MR scan room and the signal passed through the filter panel to isolate the noise from outdoor of MR scan room to control the laser module stimulating the acupoint K1 on the sole of left foot.

**Figure 1:**
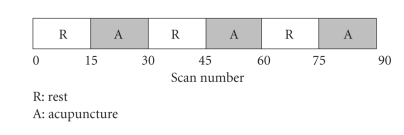
Laser acupuncture paradigm design. The paradigm was block design with two conditions (Laser irradiation: A; Laser off: R) and each block was lasted 1-minute (15 scans). Total scan time was 6 minutes (90 scans).

The fMRI scanning consisted of two runs for MW and CW stimulation types: one run applying placebo stimulation (no stimulation laser diodes turned off) and another run with laser stimulation. The order of the two runs and the switching on/off of the laser were unknown to the participant. The laser stimulation module was set up before the first run and kept in exactly the same position for all runs. During the laser stimulation, the laser light was alternately switched on and off in a typical fMRI experimental design block. For the duration of 15 fMRI scans, the laser light was alternately switched on (condition A) and off (condition R), this being controlled by hand. The measurement on a single scan took 4 seconds. A total of 90 scans were acquired (duration: 6 minutes, condition sequence: RARARA, Figure 1). Three dummy scans were acquired previous to the experimental time series to allow for magnetic saturation.

The fMRI data were processed with Statistical Parametric Mapping software (SPM2, http://www.fil.ion.ucl.ac.uk/spm/software/spm2/). After realignment, the images were normalized to the Montreal Neurological Institute (MNI) space and then smoothed spatially using an 8 mm × 8 mm × 8 mm Gaussian kernel. The smoothed data were analyzed voxel by voxel for group analysis. Statistical inferences were drawn on the basis of the general linear model as it was implemented in SPM. Linear contrasts were calculated for the comparisons between conditions. Second level group analysis was executed with a random-effects model across all experimental sessions for each condition. We designed six contrasts for analyses as follows: CW versus REST; MW versus REST; Placebo versus REST; (CW-REST) versus (Placebo-REST); (MW-REST) versus (Placebo-REST); (CW-REST) versus (MW-REST). And the conjunction analysis was performed for CW and MW conditions. To achieve significant activation at higher level comparisons between these conditions, we used a lower threshold of *P* < .002 uncorrected with 20 contiguous voxels for the comparison of (MW-REST) versus (Placebo-REST), and others were applied at a threshold of *P* < .01 uncorrected with 20 contiguous voxels. 

### 2.1. Statistical Analysis

Processing of fMRI data was done using Statistical Parametric Mapping 2 (SPM2, The Welcome Department of Cognitive Neurology, University College London, UK), and the preprocessing were realignment, coregistration, normalization of Talairach space, and smoothness with FWHM of 8 mm.

In this experiment, we are concerned with making statistical inferences from functional magnetic resonance imaging studies involving multisubject. The majority of early studies in neuroimaging combined data from multiple subjects using a “Fixed-Effects” (FFX) approach.

This methodology only takes into account the within-subject variability. It is used to report results as case studies and shows the “average effect in the group.” If we want to make formal inferences about the population from which the subjects are drawn, we have to use the Random-Effect (RFX) analysis. In neuroimaging, RFX is implemented using the computationally efficient summary-statistic approach. In the first level, fix the model for each subject using different General Linear Models (GLMs) for each subject. Then, define the effect of interest for each subject with a contrast vector. Finally, feed the contrast images into each second level GLM that implements a one-sample *t*-test. The analysis of multisubjects in this experiment was applied RFX analysis (uncorrected, *P* < .006).

## 3. Results

The group analyses for the laser stimulation versus rest or placebo show the same activation areas, and there are no significant activations for placebo versus rest because stimulation laser diodes were turned off during placebo condition. (C. M. Siedentopf et al.) The result of conjunction analysis for CW and MW conditions showed no significant activation. There are no significant deactivations for all group analyses. Thus, the group analyses were just displayed (CW-REST) versus (Placebo-REST), (MW-REST) versus (Placebo-REST) and (CW-REST) versus (MW-REST), respectively (see Tables 1–3). 

### 3.1. Comparison of (CW-REST) and (Placebo-REST)

**Figure 2:**
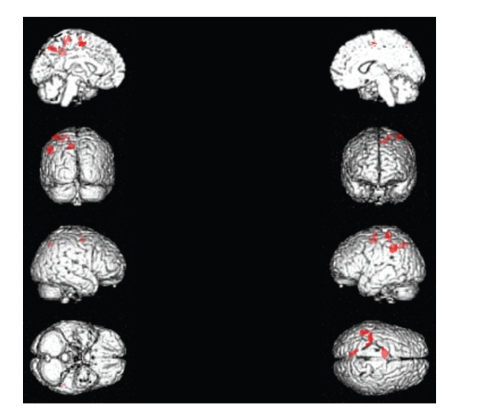
Significant activations for continued wave stimulation included the inferior parietal lobule, the primary somatosensory cortex, and the precuneus of left parietal lobe. Medial and Superior frontal gyrus of left frontal lobe.

Significant activations for continued wave stimulation were found within the inferior parietal lobule (IPL) of left parietal lobe, the left postcentral gyrus (BA 1, 2), the left precuneus (BA 7), the medial frontal gyrus of left parietal lobe, and left superior frontal gyrus (BA 6) (Table 1, Figure 2).

### 3.2. Comparison of (MW-REST) and (Placebo-REST)

**Figure 3:**
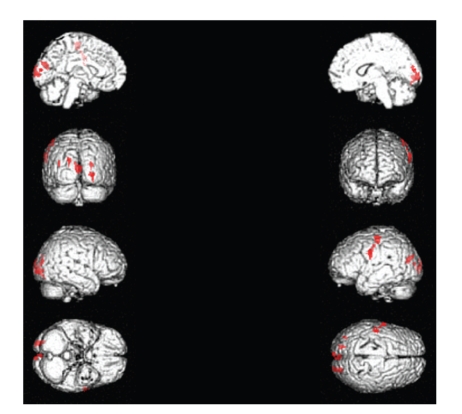
Significant activations for modulated wave stimulation included the primary motor cortex and middle temporal gyrus of left hemisphere and bilateral cuneus.

Significant activations for modulated wave stimulation were found within the precentral gyrus of left frontal lobe, the right inferior frontal gyrus, the left postcentral gyrus (BA 3), the middle temporal gyrus of left temporal lobe, the left cuneus (BA 18), and the right cuneus (Table 2, Figure 3).

### 3.3. Comparison of (CW-REST) and (MW-REST)

**Figure 4:**
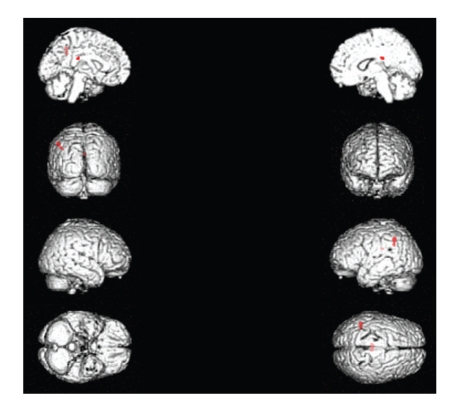
Significant activations for continued wave stimulation versus modulated wave stimulation included the left inferior parietal lobule (IPL) (BA 40) and the left supramarginal gyrus (BA 40).

Significant activations for continued wave stimulation versus modulated wave stimulation were found within the left inferior parietal lobule (IPL) (BA 40) and the left supramarginal gyrus (BA 40) (Table 3, Figure 4).

## 4. Discussion

Our study is the first report to compare the brain's hemodynamic response of MW with those of CW LLLT. This experimental study was designed to examine whether different laser acupuncture stimulation modes with the same acupoint arouse distinct brain activations. The results indicate that even if we stimulated the same acupoint with the same laser beam but modified it with a different stimulation mode, the human brain functional MRI revealed distinguishing activation.

Most articles pertaining to acupuncture researches [4–11, 22, 25] are focused on the pain release and brain activation occurring in the relevant area. In our experiment design, we stimulated the K1 acupoint. The results of our study showed most activation areas were related to attention and memory tasks, and are described as follows.

### 4.1. Activation Areas Related with Attention

First, the activation areas involved with the attention mechanism are superior frontal gyrus and inferior frontal gyrus. Rushworth et al. [26] report that superior frontal gyrus is involved in the selection of action sets. The present results demonstrate that the right inferior frontal gyrus is significantly activated during CW stimulation. The same region is involved in attention mechanisms [27] and response selection [28]. A small number of functional imaging studies have reported higher-order decision-making activity in inferior parietal lobe [29].

### 4.2. Activation Areas Related with Memory Tasks

Second, some activation regions presented in this study are concerned with memory tasks, such as parahippocampal gyrus (BA 19), inferior parietal lobule (BA 40), posterior parietal cortex, precuneus [30], and middle frontal gyrus [31]. Wagner et al. [30] point out that parietal cortex emphasizes space-based attention and motor intention, and propose that parietal cortex, including inferior parietal lobule precuneus and postcentral gyrus of parietal lobe, contributes to episodic memory retrial. Similarly, McCarthy et al. [31] also find that the spatial working memory task preferentially activated the middle frontal gyrus in the right hemisphere.

### 4.3. The Integrated Tasks Concerned with Precuneus

As the result shown, precuneus is one of the brain-activation areas by K1 acupoint stimulated in our findings. Recently, the discoveries of functional imaging suggest a central role for the precuneus in the wide spectrum of highly integrated tasks, including visuospatial imagery, episodic memory retrieval, and self-processing operations. Furthermore, precuneus and the surrounding posteromedial area display the highest resting metabolic rates and are characterized by transient decreases in the tonic activity during engagement in non-self-referential goal-directed actions (default-mode of brain function). Therefore, it has recently been proposed that precuneus is involved in the interwoven network of the neural correlates of self-consciousness, and is engaged in self-related mental representations during rest. This hypothesis is consistent with the selective hypometabolism in the posteromedial cortex reported in a wide range of altered conscious states, such as sleep, drug-induced anesthesia, and vegetative states [32].

To summarize, stimulating K1 could arouse the memory, attention, and the self-consciousness-related active area of the brain. This may provide evidence for revalidating the theory of traditional Chinese medicine. Based upon the ancient TCM book, Nan Jing (Classic of Difficulties), the acupoint, K1 (Yongquan), is one of the Well (Jing) points. At this point, the channel of acupuncture is at its most superficial and thin state. Thus, it has a particular dynamic effect when needled. The energy is at its most unstable state here, so that it can be easily and readily influenced and changed. According to chapter 68 of the Nan Jing, the Well points are used to treat irritability, mental restlessness, and anxiety. The Well points have a particularly strong effect on the mental state [33].

### 4.4. Acupuncture for Schizophrenia

Acupuncture has been used in China to treat mental health disorders, including schizophrenia, for more than 2000 years. As we know, schizophrenia is a psychotic disorder that alters patients' cognitive function, particularly affecting episodic memory and attention capacities [34]. According to our findings, LLLT on K1 acupoint could arouse brain activation relative to memory and attention capacities. Following this finding, we continued to explore whether the activation areas were concerned with schizophrenia. Shenton et al. [35] review hundreds of MRI findings in schizophrenia. Contrasting with their results, inferior parietal lobule is abnormal in schizophrenia. This region was activated in this experiment. Kindermann et al. [36] examine the function of spatial working memory among older patients with schizophrenia that demonstrate an aberrant pattern of brain response. Compared with their results, postcentral gyrus of parietal lobe is abnormal in schizophrenia. Hof et al. [37] demonstrate the existence of a significant decrease in the total number of oligodendrocytes in layer III of superior frontal gyrus in schizophrenic cases compared with control cases. Recently, Garrity et al. [38] demonstrated that the default mode functional connectivity altered in schizophrenia in many areas including superior frontal gyrus, middle frontal gyrus, and precuneus. All of these regions were activated during this experiment. It implied that LLLT on the K1 acupoint might have some effect on the attention and memory function. This effect would open a wide possibility for future schizophrenia therapy. On the basis of the systematic reviews from Rathbone, Lee and their colleagues [39, 40], the study approach with fMRI has never been reported, and it still needs more evidence and clinical testing to be proven.

### 4.5. Possible Mechanism of Laser Stimulation

Comparing EA fMRI with different frequency stimulation studies [11, 22], our result had a similar indication. All of the frequency stimulations arouse a distinct brain area in a different stimulation mode, even though the physical mechanics are different in laser and electrical stimulation. Han [21] demonstrates that neuropeptide release is frequency dependent on peripheral electrical acupuncture stimulation, and has conducted a series of experiments to analyze the possible neural pathways responsible for the frequency-specific release of different kinds of opioid peptides in rats.

Unlike electrical stimulation that directly fires the neural activation, low-level laser irradiation markedly improves the local blood flow [12, 23]. Litscher demonstrates that the laser acupuncture not only changes the peripheral blood flow [41] but also affects cerebral blood flow velocity [42]. According to Wang's resonance theory, Peripheral hemodynamic resistance can be analyzed by the arterial pulse waveform [43]. External stimulation, such as acupuncture, perturbing peripheral resistance, will redistribute the hemodynamic property of the circulation system [44, 45]. For the fMRI studies with laser acupuncture, Siedentopf et al. [13, 15] suggest the mechanism is not only based upon afferent sensory information processing. We propose that it might also result from hemodynamic weak-coupling resonance [43]; thus, a different stimulation mode applied to certain body sites can affect the blood oxygen level of a specific brain area. Interestingly, both the channel of acupuncture and the pulse diagnosis are indicated with the same Chinese character, “mai,” in the TCM.

### 4.6. Limitation

The limitation in this preliminary study should be addressed in future studies. Due to the methodological problems in fMRI studies, the activation of primary somatosensory cortex and primary motor cortex may result from the spontaneous fluctuation of resting state during 1-munite block. According to the investigation of frequency specificity in resting fMRI [19], 1-munite block length (0.008 Hz) should be much less functional connectivity in sensorimotor system than 30 s-block (0.016 Hz). Even though it had been considered the impact of resting state activity [46], primary somatosensory cortex and primary motor cortex were still aroused in this experiment.

## 5. Conclusion

**Figure 5:**
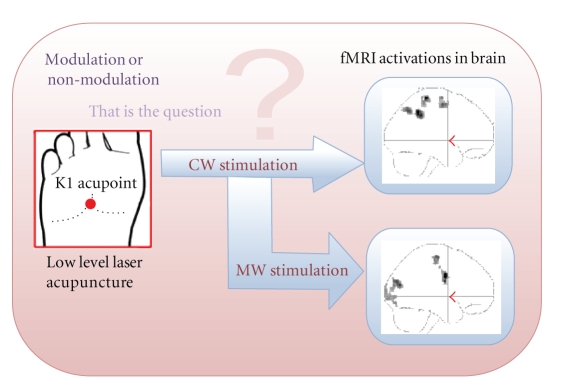
The same K1 acupoint irradiation with modulated and nonmodulated low-level laser aroused different activations in brain. (CW: continued wave; MW: 10 Hz modulation).

This study presented the functional brain mapping with the two stimulation modes of LLLT applied on the K1 acupoint. Attention- and memory-task-related brain regions were activated during LLLT on the K1 acupoint. Different brain regions rearranged the blood oxygen levels during different stimulation modes, even though the LLLT was applied to the same acupoint (Figure 5). The results imply that the mechanism of acupuncture is not only based upon afferent sensory information processing, but also relates to the hemodynamic property altered during external stimulation.

## Figures and Tables

**Table 1:** Activation clusters of continued-wave laser stimulation versus placebo group result^a^.

Laser stimulation type	Anatomical Locations	*P*-value	*T*-value	Cluster size^b^	MNI-coordinates of max value		
					*x*	*y*	*z*
Continued wave	L Parietal Lobe, Inferior Parietal Lobule	.001	4.37	144	−48	−46	44
	L Parietal Lobe, Postcentral Gyrus, BA 1	.001	4.26	123	−42	−34	66
	L Parietal Lobe, Postcentral Gyrus, BA 2						
	L Parietal Lobe, Precuneus, BA 7	.002	3.63	98	−12	−62	46
	L Frontal Lobe, Medial Frontal Gyrus	.002	3.59	173	−6	−8	56
	L Frontal Lobe, Superior Frontal Gyrus, BA 6						

^a^Activation areas of random effects analysis for clusters which surpassed a threshold of *P* < .01 uncorrected, and degrees of freedom = 11.

^b^Each voxel size = 2∗2∗2 mm^3^, and spatial extent threshold >20 voxels.

**Table 2:** Activation clusters of 10 Hz-modulation laser stimulation versus placebo group result^a^.

Laser stimulation type	Anatomical Locations	*P*-value	*T*-value	Cluster size^b^	MNI-coordinates of max value		
					*x*	*y*	*z*
Modulated wave	L Frontal Lobe, Precentral Gyrus	.000	6.45	89	−60	−4	32
	L Parietal Lobe, Postcentral Gyrus, BA 3						
	L Temporal Lobe, Middle Temporal Gyrus	.000	5.52	25	−36	−76	18
	L Occipital Lobe, Cuneus, BA 18	.000	5.28	66	−18	−80	22
	R Occipital Lobe, Cuneus	.000	4.49	31	24	−86	16

^a^Activation areas of random effects analysis for clusters which surpassed a threshold of *P* < .002 uncorrected, and degrees of freedom = 11.

^b^Each voxel size = 2∗2∗2 mm^3^, and spatial extent threshold >20 voxels.

**Table 3:** Activation clusters of continued-wave versus 10 Hz-modulation laser stimulations group resultl^a^.

Laser stimulation type	Anatomical locations	*P*-value	*T*-value	Cluster size^b^	MNI-coordinates of max value		
					*x*	*y*	*z*
Continued wave versus	L Parietal Lobe, Inferior Parietal Lobule, BA 40	.004	3.27	47	−46	−46	44
modulated wave	L Parietal Lobe, Supramarginal Gyrus, BA 40						

^a^Activation areas of random effects analysis for clusters which surpassed a threshold of *P* < .01 uncorrected, and degrees of freedom = 11.

^b^Each voxel size = 2∗2∗2 mm^3^, and spatial extent threshold >20 voxels.
